# BlpU is a broad-spectrum bacteriocin in *Streptococcus thermophilus*

**DOI:** 10.3389/fmicb.2024.1409359

**Published:** 2024-07-16

**Authors:** John A. Renye, George A. Somkuti, Phoebe X. Qi, Dennis H. Steinberg, Michael J. McAnulty, Amanda L. Miller, Giselle K. P. Guron, Adam M. Oest

**Affiliations:** Dairy and Functional Foods Research Unit, Eastern Regional Research Center, Agricultural Research Service, United States Department of Agricultural, Wyndmoor, PA, United States

**Keywords:** *Streptococcus thermophilus*, bacteriocin, quorum sensing, blp operon, thermophilin

## Abstract

*Streptococcus thermophilus* strain B59671 naturally produces thermophilin 110, a broad-spectrum bacteriocin encoded within the bacteriocin-like peptide (*blp*) gene cluster, and thermophilin 13 from a separate chromosomal locus. Analysis of the *blp* gene cluster revealed two genes, *blpU* and *blpK*, as potentially encoding bacteriocins. Deletion of *blpK* from the B59671 chromosome did not result in a loss of antimicrobial activity against either *S. thermophilus* ST113 or *Pediococcus acidilactici* F. A deletion mutant of *blpU* could not be generated in B59671, but the mature BlpU peptide obtained through overexpression in *E. coli* BL21 or chemical synthesis inhibited the growth of *S. thermophilus* strains, *Streptococcus mutans* UA159, *P. acidilactici* F, and *Listeria innocua* GV9 L-S, evidencing as a broad-spectrum bacteriocin that does not require modification for activity. This study also showed that the transcription of *blpU* was approximately 16-fold higher in B59671 than in an induced culture of *S. thermophilus* LMD-9, which produces a *blp*-encoded bacteriocin. The increased expression of BlpU in B59671 may explain the unique antimicrobial spectrum associated with this strain. Additionally, it was shown that a *blpC* deletion mutant of B59671, which prevents expression of BlpU and BlpK, inhibited the growth of other *S. thermophilus* strains and *Bacillus cereus*, suggesting that thermophilin 13 produced by B59671 possessed both intra- and interspecies antimicrobial activity. While this study confirmed that BlpU can function as an independent antimicrobial peptide, further studies are required to determine if BlpK can function independently as a broad-spectrum antimicrobial.

## Introduction

*Streptococcus thermophilus* (*S. thermophilus*) is a thermophilic lactic acid bacterium, commonly used as a starter culture in the production of yogurt and cheeses. Several strains of *S. thermophilus* have been reported to naturally produce antimicrobial peptides called bacteriocins. These ribosomally encoded peptides have been shown to display a narrow and/or broad spectrum of activity with the potential to inhibit the growth of spoilage and food-borne pathogenic bacteria (Villani et al., 1995; Ward and Somkuti, 1995; Marciset et al., 1997; Ivanova et al., 1998; Mathot et al., 2003; Gilbreth and Somkuti, 2005; Kabuki et al., 2007; Khalil, 2009; Rossi et al., 2013).

The genes required for the production of some thermophilins have been characterized, including thermophilin 13, a two-component bacteriocin (Marciset et al., 1997), and thermophilin 1277, a lantibiotic (Kabuki et al., 2007). Additionally, a bacteriocin-like peptide (*blp*) gene cluster that resembles the *blp* gene cluster from *Streptococcus pneumoniae* (de Saizieu et al., 2000), has been identified in several strains of *S. thermophilus* and shown to encode functional bacteriocins (Hols et al., 2005; Fontaine and Hols, 2008; Somkuti et al., 2015; Renye et al., 2019). The *blp* gene cluster in *S. thermophilus* was found to contain a three-gene operon (*blpABC*) that encoded the components of an ABC-transporter (*blpAB*) required for processing and secretion of a mature 30-mer quorum-sensing induction peptide (BlpC30), encoded by *blpC* (Fontaine et al., 2007). Immediately downstream of the *blpABC* operon, was a two-gene operon (*blpRH*) that encoded a histidine kinase (BlpH) and a response regulator (BlpR) required for sensing the BlpC30 induction peptide and inducing the expression of other genes within the cluster (Blomqvist et al., 2006). The remaining components of each gene cluster differ among strains and contain genes believed to encode bacteriocins based on the presence of a double glycine leader sequence within the small proteins. In strain LMD-9, the potential bacteriocin genes included *blpD*, *U*, *E*, and *F* (Hols et al., 2005); and in strains ST106 and ST109 these genes included *blpD*, *U*, and *K* (Somkuti et al., 2015; Renye et al., 2019).

Strains LMD-9 and ST106 required the addition of a synthetic BlpC30 to the growth medium to induce bacteriocin production (Fontaine et al., 2007). However, strain ST109 was capable of naturally producing its broad-spectrum bacteriocin (Renye et al., 2019). The molecular mechanism that prevents strains, such as LMD-9 and ST106, from naturally producing their respective bacteriocins remains uncharacterized. Other strains, including LMG81311 and CNRZ1066, have been shown to possess a truncation within BlpB which prevents them from producing bacteriocins, even in the presence of exogenous BlpC (Hols et al., 2005). Further analysis of LMD-9 revealed that BlpD alone was sufficient to inhibit the growth of most target bacteria, with BlpU, E, and F identified as accessory peptides that broaden the activity spectrum of BlpD (Fontaine and Hols, 2008). The presence of BlpD in strains ST106 and ST109 suggests it is also the primary bacteriocin within these strains (Somkuti et al., 2015; Renye et al., 2019). Additional variations within this gene cluster have been reported (Rossi et al., 2013), and more may arise as the number of fully sequenced strains of *S. thermophilus* increases.

Previous work from our laboratory described the natural production of an anti-pediococcal bacteriocin, thermophilin 110, by *S. thermophilus* B59671 (Gilbreth and Somkuti, 2005), and showed that bacteriocin production was regulated by BlpC (Renye and Somkuti, 2013). *In silico* analysis of the sequenced B59671 genome later revealed the presence of the thermophilin 13 gene cluster in addition to the *blp* gene cluster (Renye et al., 2017; Salini et al., 2023). McAnulty et al. (2024) showed that thermophilin 13 was expressed in B59671, and transcription increased when BlpC was deleted (STB59671C^−^), suggesting cross-talk between the bacteriocin regulatory systems. STB59671C^−^ still inhibited the growth of *S. thermophilus* 113, confirming the intraspecies antimicrobial activity of thermophilin 13. However, when the genes encoding thermophilin 13 (*thmA/B*) were knocked-out, the strain still inhibited the growth of *P. acidilactici* F and *Cutibacterium acnes* ATCC6919, suggesting that interspecies bacteriocin activity was due to the production of thermophilin 110.

The purpose of this study was to determine which genes within the *blp* gene cluster encoded the active bacteriocin through the use of gene knockout mutants, and the overexpression or synthesis of targeted peptides for use in *in vitro* antimicrobial assays.

## Materials and methods

### Bacterial strains and growth medium

*S. thermophilus* strains B59671 (NRRL, Agricultural Research Service Culture Collection, NCAUR, USDA, Peoria, IL), ST109, ST113, ST128 (in-house collection), LMG18311, LMD-9, and *Streptococcus salivarius* K12 (ATCC BAA1024) were grown in tryptone-yeast extract-lactose (TYL) broth at 37°C. *Pediococcus acidilactici* strain F (gift from B. Ray, University of Wyoming) and *Lactobacillus helveticus* 1942 (in-house collection) were grown in deMan, Rogosa, and Sharpe (MRS) medium. *Escherichia coli* strains DH5α and BL21, *Enterococcus faecalis* VA797, *Enterococcus faecium* H3, *Bacillus cereus* ATCC1457, and *Listeria innocua* GV9-LS were grown in Brain Heart Infusion (BHI) medium, and *Streptococcus mutans* strains GS-5 and UA159 were grown in Todd Hewitt Broth (THB) at 37°C. All strains were grown aerobically with *E. coli*, *B. cereus* and *L. innocua* broth cultures agitated at 200 rpm. Gene expression within the LMD-9 *blp* locus was induced by adding a final concentration of 250 ng/mL of synthesized BlpC30 (stock solution 1 mg/mL in H_2_O) to the growth medium. BlpC30 (30 mer induction peptide) was prepared by microwave-assisted solid phase peptide synthesis with Fmoc-protected amino acids, using a CEM Liberty synthesizer (CEM Corp., Matthews, NC). The purified peptide was analyzed by HPLC and the molecular mass was confirmed by MALDI-TOF MS.

### Bacteriocin activity

Bacteriocin activity was measured using an agar diffusion method (Gilbreth and Somkuti, 2005). Cultures of *S. thermophilus* B59671 and LMD-9 were grown to an OD_660_ of ~0.8 in TYL and pelleted at 10,000×*g* for 10 min at 4°C for collection of cell-free supernatants (CFS). CFS (~50 μL; ~320 AU/mL) was loaded into precast wells in an agar medium (1.5%) seeded with the target bacteria (0.5% v/ v). Bacterial targets included: *S. thermophilus* ST109, ST113 (positive control strain), 128, and LMG18311, *S. salivarius* K12 in TYL; *P. acidilactici* (positive control strain) and *L. helveticus* in MRS; *B. cereus, E. faecalis* and *E. faecium* in BHI; and *S. mutans* GS-5 and UA159 in THB. Plates were allowed to equilibrate at 4°C overnight, and then incubated at 37°C and examined for the appearance of zones of inhibition. ST113 was routinely run as a negative control to show that inhibition zones were not due to acid production.

### Scanning electron microscopy (SEM)

*P. acidilactici* F cells were grown overnight at 37°C, and then diluted in fresh MRS (25 fold) and grown until mid-log phase (O.D._600 nm_ = 0.5). Cells were harvested by centrifugation (10,000×*g* for 5 min at 4°C) and resuspended in 0.1% peptone water at the same optical density. Cells were then incubated at 37°C in the absence or presence of 510 AU/mL thermophilin 110. Culture O.D. was monitored for up to 3 h, and cells were collected at various times with 50 μL of bacteria placed on acetone-cleaned 12 mm Micro-cover glass slides (Thermo Scientific, Portsmouth, NH, United States) and allowed to adhere for 30 min. Samples were covered with 2 mL of 2.5% glutaraldehyde, (Electron Microscopy Sciences, Hatfield, PA, United States) and allowed to fix for 30 min. Samples were rinsed twice (30 min each) with 2–3 mL 0.1 M imidazole, (Electron Microscopy Sciences, Hatfield, PA, United States), followed by washing with 50, 80, and 90% ethanol (The Warner-Graham Company, Cockeysville, MD, USA) at 30-min intervals (2–3 mL each). The samples were then washed three times with 2 mL of 100% ethanol (30-min per wash) before critical point drying. The samples were stacked in a wire basket, separated by cloth, and placed in a *Critical Point Drying Apparatus* (Denton Vacuum, Inc., Cherry Hill, NJ, United States), using liquid carbon dioxide (Welco Co, Allentown, PA, United States) for approximately 20 min. The samples were mounted on stubs and sputter gold coated for 1 min (EMS 150R ES, EM Sciences, Hatfield, PA). Samples were then viewed with an FEI Quanta 200 F Scanning Electron Microscope (Hillsboro, OR, United States) with an accelerating voltage of 10 KV in high vacuum mode.

### Sequencing of the *blp* gene cluster

Sequencing of the *blp* gene cluster in *S. thermophilus* B59671 was performed by chromosome walking using purified genomic DNA as a template. The initial PCR primers were designed based on the nucleic acid sequence of LMD-9, corresponding to the 5′ end of *blpA* (5′-ATGTTTCGTTTTCGTAGG-3′) and the 3′ end of *blpX* (5′-AATTTTTACGATTCATTT-3′). After each round of sequencing, a new primer was designed based on the obtained sequence and walking was continued until the entire gene cluster was sequenced in both directions. Nucleic acid sequencing was performed using a 3730 PRISM^®^ DNA analyzer (Applied Biosystems, Foster City, CA) with the ABI PRISM Big Dye terminator cycle sequencing reagent. Sequences were analyzed and contigs were created using Sequencher 4.9 (Gene Codes Corp, Ann Arbor, MI). Open Reading frame (ORF) identification and promoter analysis were performed using Clone Manager software (Sci-ed Software, Cary, NC).

### Real-time PCR analysis

*S. thermophilus* strains were grown in TYL broth until the culture reached an OD_600_ between 0.8 and 1.0. RNA was extracted using the RiboPure™-Bacteria kit (Ambion-Life Technologies, Grand Island, NY). To eliminate any residual DNA, RNA was treated with DNase I (Ambion) for 30 min at 37°C. RT-PCR was performed using a Lightcycler^®^ 96 (Roche Diagnostics Corp., Indianapolis, IN). Primers for *blp*C*, blp*D*, blp*U and 16S rRNA (control) are listed in Table 1. Cycling conditions were selected as the following: 40 cycles of 95°C for 30 s, 45°C for 30 s, and 60°C for 30 s. The melt curve analysis was performed from 60 to 95°C with fluorescence readings taken continuously after a 1% temperature increase. cDNA synthesis was performed with Transcriptor Universal cDNA Master (Roche) with 1 μg of total RNA. RT-PCR analysis was conducted with 0.015 ng cDNA using the Fast Start Essential DNA Green Master (Roche). Lightcycler^®^ 96 software was used to determine C_q_ values and relative quantification. Results were reported from a minimum of three independent RT-PCR reactions with the 16S rRNA gene used as a reference and shown to be stably expressed.

**Table 1:** Real time PCR primers pairs.

Gene	Forward 5′-3′	Reverse 5′-3′
*blpC*	AAATGGTTTCCTAAAAGGATTCG	GAGGAATGTTATAGTCTTTTGTTGGA
*blpD*	CAGTAGGAGGACTTGGTGCAGT	CAGTATAAGGCTGCACCTACTAACA
*blpU*	CCACCAGCATGTTGCTCC	GCTAAACAAGGAGTAGCTAC
RT-PCR 16S rRNA	TACCAGAAAGGGACGGCTAA	CGCTCGGGACCTACGTATTA

### Construction of *blpK* knock-out strain

An integrative vector for knocking out *blpK* in *S. thermophilus* B59671 was constructed as previously described (Renye and Somkuti, 2013). The primers used to amplify 600 bp fragments of *blpK* flanking genes were designed as follows: *orf6* Fwd (*SpeI*): CCCACTAGTCGCTAAGGGCTTACTTGA and *orf6* Rev. (*PstI*): CCCCTGCAGGG-AAATCTTCCTCTAATT; and *ISB* Fwd (*XhoI*): CCCCTCGAGGCTTTGTTAGGTAATATC and ISB Rev. (*KpnI*): CCCGGTACCGAGGACAACATTCTCAATCG. The amplified fragments were cloned into pKS1 (Shatalin and Neyfakh, 2005) at the corresponding restriction sites, resulting in the plasmid, pSTKOK, and subsequently transformed and propagated in *E. coli* DH5α. The integrative vector pSTKOK was then electroporated into strain B59671 using a previously described protocol (Somkuti and Steinberg, 1988), with transformants selected on TYL containing 15 μg/mL erythromycin (erm) at 30°C. Transformants were sub-cultured in TYL+ erm at 37°C to allow for chromosomal integration of pSTKOK, and then sub-cultured in TYL at 30°C to allow for the second homologous recombination event to occur. A B59671 mutant with *blpK* replaced by a kanamycin resistance marker was confirmed by PCR using the primers: *orf5*Fwd: GAAAACAAAGATATGTATATTCGCCTA and ISB2Rev: AACGTTTTCAAGAGCGCAAT. *S. thermophilus* transformants were maintained in TYL containing 150 μg/mL kanamycin.

### Heterologous expression of mature BlpU peptide

The nucleic acid sequence (162 base pairs) corresponding to the mature BlpU peptide (excluding the 23 amino acid leader peptide) was amplified from *S. thermophilus* B59671 with the primers: BlpU Fwd (*BamHI*): ATAGGATCC*ATCGAAGGTCGT*GGATGTAGCTGGGGAGGT (nucleotides encoding the Factor Xa recognition sequence (isoleucine-glutamic acid-glycine-arginine) are italicized) and BlpU Rev. (*XhoI*): ATCTCGAGTCACCACCAGCATGTTGCTC. PCR was performed using the high-fidelity *Pfx Taq* polymerase (Life Technologies, Grand Island, NY); and the resulting DNA fragment was cloned into pGEX 6P-1 (GE Healthcare Life Sciences, Piscataway, NJ) at the corresponding restriction sites, downstream of the isopropyl β-D-1-thiogalactopyranoside (IPTG) inducible promoter. Cloning was confirmed by PCR using primers specific to *blpU*.

The plasmid was transformed into *E. coli* BL21 for recombinant expression of the fusion protein. *E. coli* containing pGEX 6P-1/blpU was grown overnight in BHI broth containing 100 μg/mL ampicillin (amp) at 37°C under agitation at 200 rpm. The culture was then diluted 1:100 into 100 mL BHI amp and grown at 37°C until the optical density (OD_600_) reached 0.8–1.0, at which point IPTG was added at a final concentration of 0.1 mM. The culture was incubated for an additional 6 h, after which cells were collected by centrifugation at 10,000×*g* for 10 min, washed twice with phosphate-buffered saline (PBS) prior to resuspension in 20 mL PBS, and lysed by sonication (10 pulses of 30 s) using a microtip cell disruptor (Heat Systems Ultrasonics, Farmingdale, NY, United States). The lysate was centrifuged to remove cellular debris, then mixed with 1x bed volume of a 50% slurry glutathione Sepharose 4B resin (GE Healthcare) and incubated at 25°C for 2 h and 4°C overnight. The GST-BlpU fusion protein (~31 kDa) was visualized on a 12% Bis-Tris SDS-PAGE with Simply Blue™ SafeStain (ThermoFisher Scientific, Waltham, MA), was eluted from the Sepharose 4B resin as described by the manufacturer and dialyzed (12–14 kDa molecular weight cut off) against Factor Xa digestion buffer (20 mM Tris/HCl-100 mM NaCl-2 mM CaCl, pH 8.0) at 4°C overnight. The solution within the dialysis bag was recovered and analyzed by SDS-PAGE and then digested with 50 μL (1 mg/mL) of Factor Xa protease. The digested mixture was analyzed by 12% Bis-Tris SDS-PAGE with silver nitrate staining and analyzed for antimicrobial activity against *P. acidilactici* using a gel overlay method (Gilbreth and Somkuti, 2005).

### Synthesis of BlpU

The mature BlpU peptide (53 aa: GCSWGGFAKQGVATGVGNGLRLGIKTRTWQGA-VAGAAGGAIVGGVGYGATCWW) was synthesized and purified by high-performance liquid chromatography (Lifetein LLC, South Plainfield, NJ) to a purity of 85.9%. The peptide was dissolved in dimethyl sulfoxide (DMSO) at a stock concentration of 4.6 mg/mL. Further dilution to a working stock of 100 μg/mL was carried out in ddH_2_O. Antimicrobial activity was determined in a well-diffusion assay using 50 μL of the working stock solution.

## Results

### Characterization of the *S. thermophilus* B59671 *blp* gene cluster

**Figure 1:**
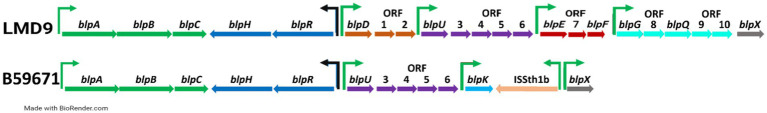
Map of the *Streptococcus thermophilus* bacteriocin-like peptide (*blp*) gene cluster in strains LMD-9 (top) and B59671 (bottom). Homologous gene or operons are grouped by color, and constitutive promoters (black arrows) and BlpC30 inducible promoters (green arrows) are shown. Gene nomenclature was taken from Fontaine et al. (2007), and gene maps were created with BioRender.com.

Chromosome walking was used to sequence the *blp* gene cluster within strain B59671, leading to a single 10.7 kb contig shown to contain 14 open reading frames (ORFs) (Figure 1). The resulting contig was aligned with the circularized whole genome sequence (WGS) of strain B59671 (Renye et al., 2017) to identify the location of the *blp* locus and confirm the accuracy of the WGS sequencing results. As reported for other *S. thermophilus* strains, the three-gene operon containing homologs of *blpA, blpB*, and *blpC* was directly adjacent to the two-gene operon encoding for the histidine kinase (BlpH) and response regulator (BlpR) required for inducing the expression of genes within the *blp* locus (Bolotin et al., 2004; Fontaine et al., 2007; Sun et al., 2011; Kang et al., 2012). When compared with the *blp* locus in LMD-9 (Accession number: CP000419), it was determined that the predicted proteins, BlpR and BlpH, differed by 7 and 11 amino acids, respectively, in B59671.

In strain B59671, the region between the *blpRH* operon and *blpX* contained two ORFs which encode putative antimicrobial peptides based on the presence of a double glycine (GG) leader sequence. The predicted mature peptide encoded by *blpU* showed 100% identity with the corresponding peptides in LMD-9, LMG18311 (Accession number: CP000023) and ST106, but differed with the peptide expressed in ST109 by a single amino acid substitution, valine to glycine at the N-terminus (Renye et al., 2019). In all strains *blpU* was part of a two-gene operon with *orf3*, which was predicted to encode the immunity protein required for protection against BlpU (Fontaine and Hols, 2008). The second ORF within the B59671 *blp* gene cluster was predicted to encode an 81 amino acid peptide with 98% identity to BlpK in strain CNRZ1066 (Hols et al., 2005), but differed more with the BlpK homologs in ST106 and ST109 due to the presence of a four-amino acid repeat (IGGA) insertion at positions 20–23 of the mature peptide (Renye et al., 2019).

The nucleic acid sequences upstream of the *blpABC*, *blpU-orf3*, and *blpK* operons were compared to the previously characterized *blp*-inducible promoters within the LMD-9 gene cluster (Fontaine et al., 2007). The *blpABC* promoter in strain B59671 was identical to the characterized region in LMD-9, including the presence of two imperfect direct repeats (DR), the left repeat (LR): ACCGTTTGGGACG and right repeat (RR): ACTTTTTGGGACG, shown to be essential for BlpC30-induced gene expression (Blomqvist et al., 2006). The promoters upstream regions of *blpU-orf3* and *blpK* matched the promoter region upstream of the *blpE/orf7/blpF* operon in LMD-9. This promoter sequence differed from the *blpU* promoter in LMD-9 by two nucleotides in the LR sequence: ACCATTCGGGACA (B59671) and ACTACTCGGGACA (LMD-9) (Fontaine et al., 2007).

### Bacteriocin expression in strains B59671 and LMD-9

**Figure 2:**
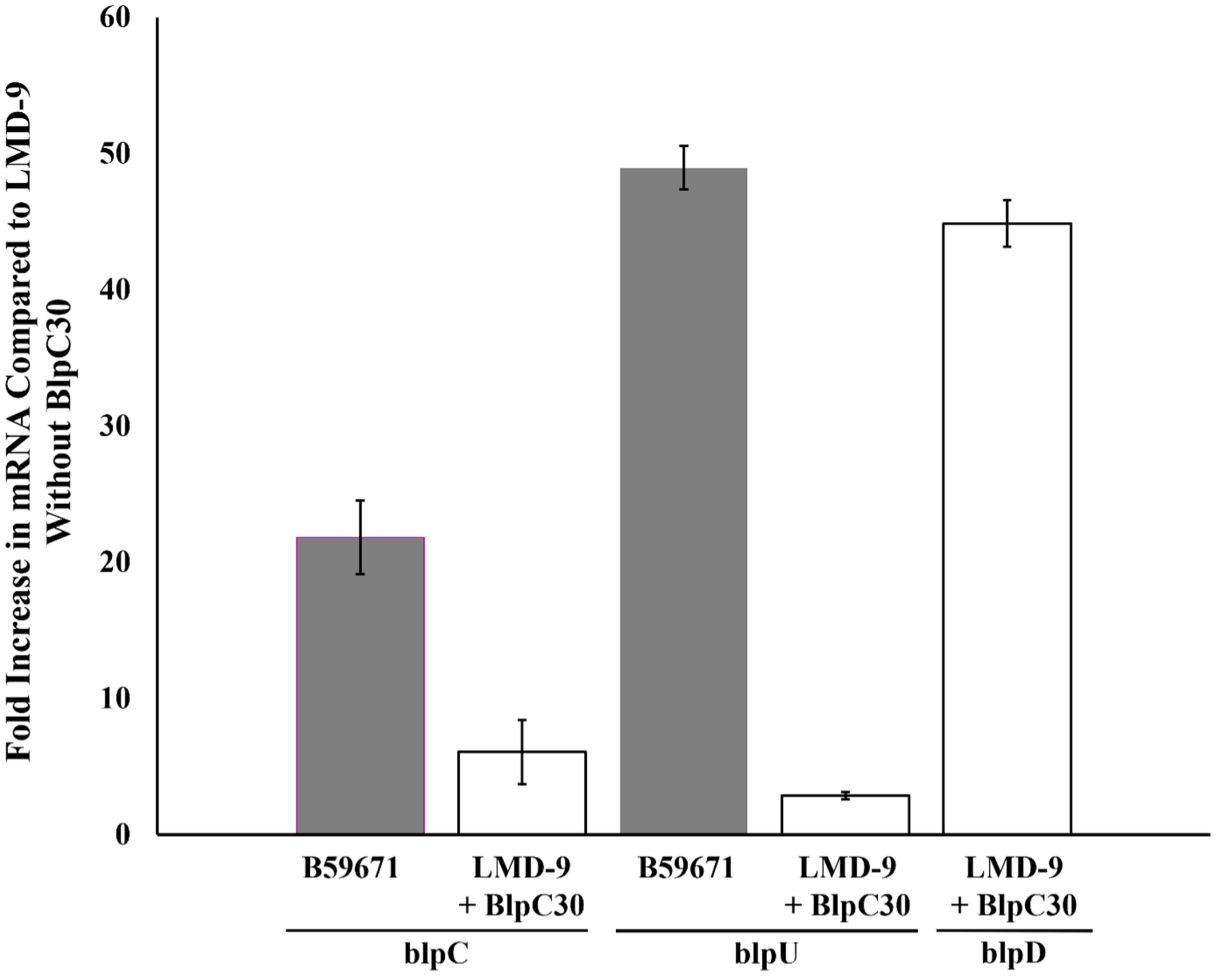
Real-time PCR analysis of *blpC* and *blpU* expression in B59671 (gray bars) and LMD-9 + (white bars); and *blpD* in LMD-9 + BlpC30 (white bar) in tryptone yeast extract (TYL) medium. Expression is presented as the fold-increase in mRNA levels as compared to the LMD-9 control (non-induced). Data are the average of at least three independent experiments (± Standard Deviation).

Previously it was reported that thermophilin 110 was naturally produced by *S. thermophilus* strain B5967; however, bacteriocin production by strain LMD-9 required induction with the BlpC quorum sensing peptide (BlpC30) at 250 ng/mL (Renye et al., 2016). Semiquantitative RT-PCR was used to compare the expression of *blp* components known to be upregulated by BlpC30 when strains B59671 and LMD-9 reached late exponential phase (OD_660_ of ~0.8). The level of *blpC* mRNA was substantially higher in B59671 when compared to both non-induced and induced cultures of LMD-9 (Figure 2), similar to what was observed when transcription levels were compared between B59671 and ST106 (McAnulty et al., 2024). Following induction with the BlpC30 peptide, BlpC expression increased by 6-fold in LMD-9, ~16-fold lower than the expression level in B59671. Transcription of the *blpU-orf3* operon was more than 45-fold higher in B59671 when compared to both induced and non-induced cultures of LMD-9 (Figure 2). Consistent with a previous report (Fontaine et al., 2007), transcription of the *blpuU-orf3* operon responded poorly to induction with BlpC30 in LMD-9 (~3-fold). However, the expression of BlpD increased by ~45 fold following induction with BlpC30 in LMD-9 (Figure 2), a level of expression similar to what was observed for BlpU in B5967. In B59671, the expression level of BlpK was comparable to that of BlpU, which was expected since their promoter regions were identical (data not shown).

### Antimicrobial activity of bacteriocins encoded within the B59671 *blp* locus

**Figure 3:**
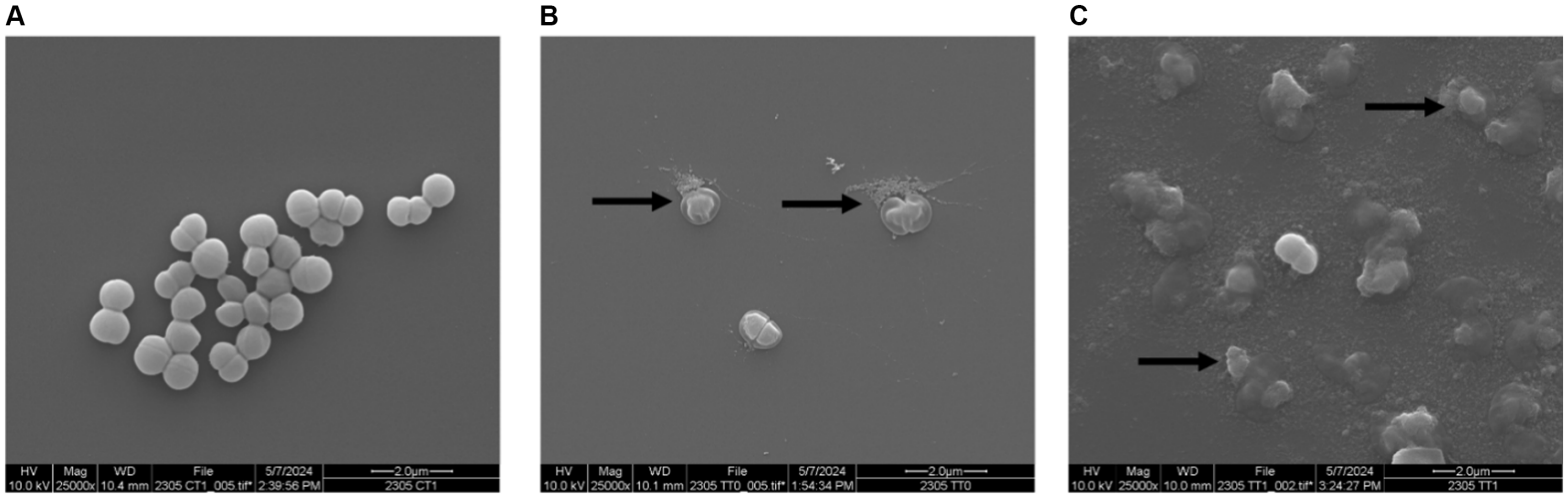
Lysis of *P. acidilactici* by thermophilin 110. *P. acidilactici* cells grown in MRS until mid-exponential phase, and then resuspended in 0.1% peptone water in the absence **(A)** or presence of 512 AU/mL thermophilin 110 **(B, C)**. SEM images were collected after *P. acidilactici* cells were in peptone water without thermophilin 110 **(A)** or exposed to thermophilin 110 for 0.5 h **(B)** or 1.5 h **(C)**, with images shown at 25,000X magnification.

Thermophilin 110 was previously reported to induce cell lysis of *P. acidilactici* (Gilbreth and Somkuti, 2005) and *Cutibacterium acnes* (Renye et al., 2023) based on a drop in culture optical density (OD_600nm_). In this study, when *P. acidilactici* cells were exposed to 512 AU/mL thermophilin 110, the optical density of the culture dropped from 1.06 (OD_600nm_) to 0.299 (OD_600nm_) by 2 h; and viable counts dropped from 8.49 log CFU/mL (T_0_) to 5.96 log CFU/mL (T_2_). Additionally, lysed cells were observed by SEM within 0.5 h (Figure 3B, arrows), with several fully lysed cells observed after 1.5 h (Figure 3C, arrows). These results confirmed the bactericidal activity of thermophilin 110.

Additionally, the activity spectrum of thermopilin 110 was confirmed using a selection of previously reported susceptible bacteria (Gilbreth and Somkuti, 2005; Renye and Steinberg, 2021), since a recent study revealed that *S. thermophilus* B59671 produces a second active bacteriocin, thermophilin 13 (McAnulty et al., 2024). Cell-free supernatants from B59671C^−^ maintained intraspecies activity against four *S. thermophilus* strains and showed broad-spectrum activity against *B. cereus* (Table 2), most likely due to the production of thermophilin 13. Antimicrobial activity was not observed against enterococci, *Lb. helveticus*, *S. mutans*, and *S. salivarius* when CFS from B59671C^−^ was tested, suggesting that these unique activities were dependent on the production of thermophilin 110.

**Table 2:** Antimicrobial activity of bacteriocins produced by *S. thermophilus* B59671.

Target strain	B59671 Parent	B59671 *blpC* Knock Out
*B. cereus* ATCC1457	+	+
*E. faecalis* VA797	+	−
*E. faecium* H3	+	−
*L. helveticus B1942*	+	−
*S. mutans* GS-5	+	−
*S. mutans* UA159	+	−
*S. salivarius* K-12	+	−
*S. thermophilus* ST109	+	+
*S. thermophilus* ST113	+	+
*S. thermophilus* S128	+	+
*S. thermophilus* LMG18311	+	+

**Figure 4:**
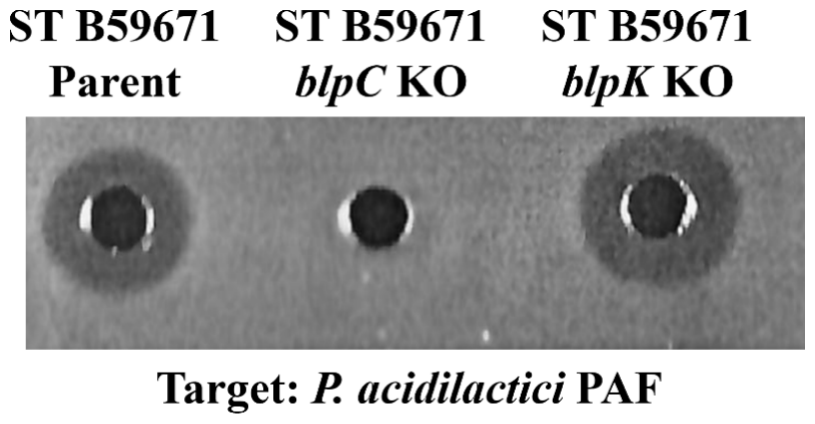
Antimicrobial activity of *S. thermophilus* B59671 and knock out mutants against *Pediococcus acidilactici* PAF. Cell free supernatants were collected after 24 h of growth and loaded into precast wells. Anti-pediococcal activity was evident by zones of inhibition around wells. (Left to Right) Well 1: CFS from ST B59671 Parent strain; Well 2: ST B59671 *blpC* knock out mutant; Well 3: B59671 *blpK* knock out mutant.

To determine which genes encoded active bacteriocins within the B59671 *blp* gene cluster, the integrative vector pKS1 was used to construct deletion mutants for both *blpU* and *blpK*. A plasmid was successfully constructed to replace *blpU* with a kanamycin resistance maker (*kan^r^*), but the knock out mutant was not obtained after several attempts (data not shown). However, the vector pSTKOK was generated and successfully used to delete the chromosomal copy of *blpK* from B59671. The mutant, *S. thermophilus* B59671K^−^, was sensitive to erythromycin and resistant to kanamycin, confirming a double crossover recombination event where *kan^r^* was inserted within the chromosome. The mutant was further confirmed by PCR analysis using primers corresponding to the genes *orf5* and *ISSB*, which flank *blpK*. Insertion of *kan*^r^ increased the PCR product size from 1.3 kB to 2.8 kB. Cell-free supernatant from an overnight culture of *S. thermophilus* B59671K^−^ (*blpK* knockout) was shown to inhibit the growth of *P. acidilactici* PAF (Figure 4, well 3), with a zone similar in size to the parent culture (well 1). However, ST B59671C^−^ did not inhibit pediococcal growth (Figure 4, well 2), validating that the active bacteriocin was encoded within the *blp* locus. These results suggested that BlpU was a bacteriocin with anti-pediococcal activity.

### Antimicrobial activity of *BlpU*

**Figure 5:**
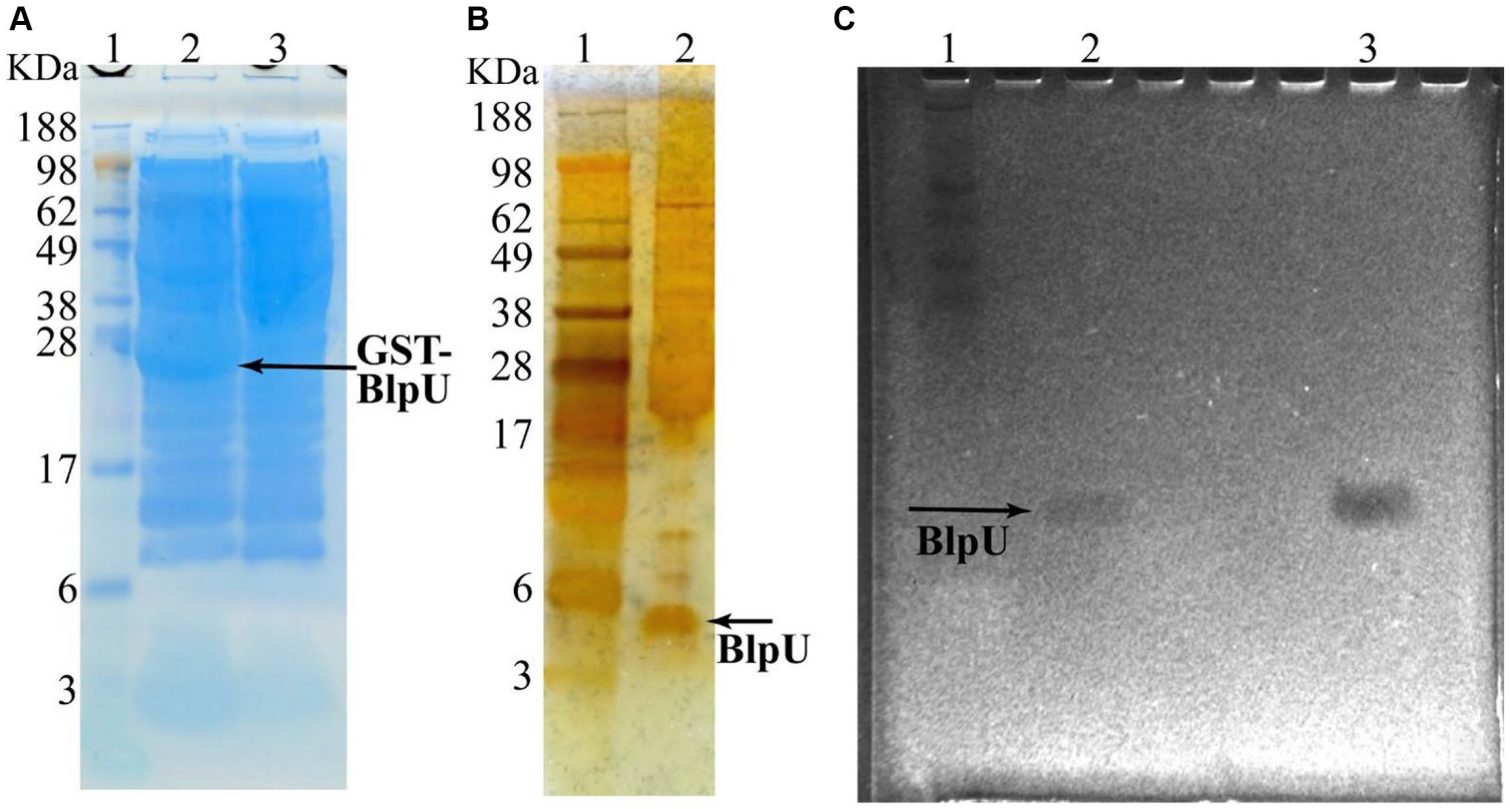
Expression of recombinant mature BlpU peptide in *E. coli* BL21. **(A)** Simply Blue stained SDS-PAGE of cell lysates from *E. coli* BL21 containing plasmid which encodes GST-BlpU fusion protein. Lane 1: Protein marker; Lane 2: Lysate from IPTG-induced cells, GST-BlpU labeled (arrow); Lane 3: Lysate from cells that were not induced (no fusion protein). **(B)** Silver nitrate stained SDS-PAGE of purified BlpU following Factor Xa cleavage. Lane 1: Protein marker; Lane 2: Lysate following Factor Xa cleavage, BlpU labeled (arrow). **(C)** Gel overlay assay showing inhibition zones with *P. acidilactici* PAF used as the target bacterium. Lane 1: Protein marker; Lane 2: Recombinant BlpU from *E. coli*; Lane 3: CFS from overnight ST B5971 culture.

Since the creation of a *blpU* knockout strain was unsuccessful, overexpression of the predicted mature peptide (without the 23 amino acid leader sequence) was demonstrated using *E. coli* BL21 as the host bacterium. The plasmid pGEX 6P-1/*blpU* was constructed to express a GST-BlpU fusion protein, which also contained a Factor Xa cleavage sequence at the N-terminus of BlpU. The recombinant gene was located downstream of an IPTG inducible promoter, and transcription was induced when the culture reached an OD_600_ ~ 0.8, with the culture incubated for an additional 6 h. After lysis of the induced *E. coli* cells, the fusion protein was recovered using 50% slurry of glutathione Sepharose 4B resin. The eluted protein was run on an SDS-PAGE gel which showed the presence of an expected fusion protein, ~31 kDa (Figure 5A, lane 2). The band corresponding to the fusion protein was not observed in uninduced cells (Figure 5A, lane 3), or in a lysate from *E. coli* BL21 without the expression plasmid, attesting that the protein band was not a host cell protein. The GST-BlpU fusion protein did not show antimicrobial activity against *P. acidilactici*, most likely due to the presence of the GST tag on its N-terminus (data not shown). After Factor Xa cleavage, a small peptide (~5 kDa) was observed by SDS-PAGE analysis after staining with silver nitrate (Figure 5B, lane 2), and a faint inhibition zone corresponding to the released peptide was observed when a washed SDS-PAGE was overlayed onto an agar medium inoculated with *P. acidilactici* (Figure 5C, lane 2). This confirmed that the GST tag must be removed to render BlpU active; and the location of the active BlpU on gel overlay corresponded to the antimicrobial peptide present in CFS from *S. thermophilus* B59671 (Figure 5C, lane 3). The inhibition zone results strongly suggested that BlpU is a broad-spectrum bacteriocin produced by this strain.

**Figure 6:**
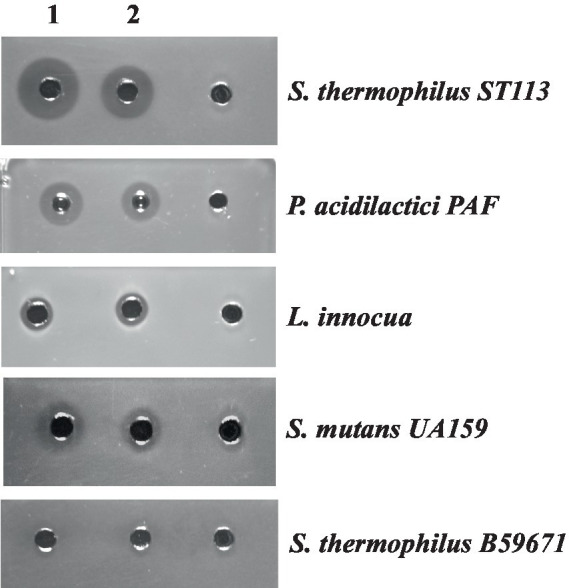
Antimicrobial activity of the chemically synthesized mature BlpU peptide against selected bacterial targets: *S. thermophilus* ST113*, P. acidilactici* PAF, *L. innocua, S. mutans* UA159 and *S. thermophilus* B59671. Wells in column 1: 50 μL of partially purified thermophilin 110 from B59671 CFS; column 2: 50 μL of the synthesized BlpU (100 μg/mL); column 3: 50 μL of the DMSO/water solution used to resuspend synthetic peptide.

To further establish that BlpU is an active bacteriocin in the absence of other components encoded within the *blp* gene cluster of *S. thermophilus* B59671, the mature peptide was chemically synthesized. The resulting peptide (purity ~86%) was resuspended in DMSO at a concentration of 4.6 mg/mL. The peptide at a working concentration of 100 μg/mL (in sterile water) was applied to inhibit the growth of *S. thermophilus* ST113, *P. acidilactici*, *L. inoccua*, and *S. mutans* (Figure 6, column 2). Inhibition zones were comparable to the zones observed using a partially purified thermophilin 110 preparation (~512 AU/mL) as a control (Figure 6, column 1). Inhibition zones were not observed around wells loaded with a diluted DMSO solution as a negative control, evidencing the activities arose from the presence of BlpU (Figure 6, column 3). *S. thermophilus* B59671 was shown to be resistant to all bacteriocin preparations.

## Discussion

Natural production of *blp* bacteriocins has only been reported in a limited number of strains, including B59671 and ST109 (Gilbreth and Somkuti, 2005; Renye and Steinberg, 2021), whereas the production of Blp bacteriocins must be induced in other strains, including LMD-9, ST106, and ST118 (Fontaine et al., 2007; Somkuti and Renye, 2015) by either overexpressing *blpC* or supplementing the growth medium with synthetic BlpC30. While BlpD has been shown to be essential for the antimicrobial activity in LMD-9 (Fontaine and Hols, 2008), the *blp* gene cluster in B59671 lacks a homolog of *blpD*. Since *blpU* and *blpK* are the only genes in the B59671 *blp* cluster that encode proteins with a conserved class II bacteriocin double-glycine motif, it is implied that BlpU and/or BlpK must be the active bacteriocin in B59671.

The *blp* gene cluster in B59671, as well as in LMD-9 and ST106, encodes a fully functional quorum sensing system, including the peptide processing and transport proteins (BlpAB), quorum sensing induction peptide (BlpC), and a histidine kinase and response regulator (BlpHR). However, strains LMD-9 and ST106 do not produce their respective bacteriocins naturally (Fontaine et al., 2007; Somkuti and Renye, 2015), most likely a result of the quorum sensing peptide, BlpC, never reaching a threshold concentration required to induce gene expression from within the *blp* loci. This study showed that transcription of *blpC* was more than 20-fold higher in B59671 than in a BlpC30-induced culture of LMD-9. A similar result was reported in a transcriptomic study of B59671 and ST106 (McAnulty et al., 2024), again suggesting the constitutive expression of BlpC was not sufficient to induce transcription of other components within the *blp* gene cluster.

In strain LMG18311, a truncation in BlpB explains why the *blp* quorum sensing system does not function, but comparative genomic studies have not established why the *blpABC* operon is expressed significantly higher in strains B59671 and ST109. The promoter sequence upstream of the *blpABC* operon is identical in strains B59671 and LMD-9, and so is the nucleic acid sequence of the *blpABC* and *blpRH* operons, which reside on opposite DNA strands. It was previously hypothesized that a leaky terminator between the *blpABC* and *blpRH* operons may allow for the formation of a double-stranded RNA molecule that could be degraded by RNase III (Lamontagne and Elela, 2004). However, it remains unclear why this would only occur in selected strains. Another possibility is that transcriptional regulators located outside the *blp* gene cluster may regulate the expression of the *blp* quorum sensing system. Cross talk via ComR (Fontaine et al., 2013), or other bacteriocin signaling peptides (*thmX*) or global transcriptional regulators within *S. thermophilus* (McAnulty et al., 2024) are potential candidates and need to be explored further.

Homologs of *blpU* are present in LMD-9, ST106, and ST109, yet these strains have not been reported to inhibit the growth of some bacterial targets sensitive to thermophilin 110, including *S. mutans*, *C. acnes* 6,919, and *P. acidilactici* F. This may be attributed to a lowered level of BlpU production in these strains. In LMD-9, BlpU was characterized as an accessory peptide that extends the activity spectrum of BlpD (Fontaine and Hols, 2008). The expression of *blpU* was induced by the presence of BlpC30, however, induction was lower than what was observed for the *blpD-orf2* or *blpE-blpF* operons in LMD-9 (Fontaine et al., 2007). Analysis of the promoter upstream regions of *blpU* and *blpK* in B59671 showed that they are identical to the promoter upstream of the *blpE-blpF* operon in LMD-9, which was the second most responsive promoter to BlpC30 induction in LMD-9 (Fontaine et al., 2007). The promoter upstream of *blpU* in other strains may limit peptide expression so that the unique antimicrobial activities associated with the production of BlpU are not observed. Additionally, strain B59671 does not possess a homolog of *blpG*, which encodes a thiol-disulfide oxidase that is required for peptide modification and subsequent antimicrobial activities described for strain LMD-9 (Fontaine and Hols, 2008). The absence of BlpG in B59671 may prevent modifications to BlpU which are detrimental to the unique antimicrobial activities described in this study.

Characterization of the antimicrobial activity associated with *S. thermophilus* B59671 is complicated by the presence of two functional bacteriocin gene clusters (Salini et al., 2023; McAnulty et al., 2024). Acquiring the ability to produce two separate bacteriocins may have provided a competitive advantage for B59671 to outcompete closely related species within its natural niche, and now may make it an ideal candidate for use in food and non-food applications. When compared to traditional antibiotics, bacteriocins have a limited spectrum of activity and their use is not expected to result in the selection of microorganisms resistant to clinically relevant antimicrobials. This has allowed bacteriocin-producing lactic acid bacteria to be investigated for improving the safety and shelf-life of foods, and as probiotics. However, potential applications for *S. thermophilus* strains as bioprotective cultures remains scarce. This is predominantly due to most strains not being able to produce their bacteriocins naturally; however, this study showed that B59671 exhibits a broad spectrum of antimicrobial activity due to the natural production of both thermophilin 110 and thermophilin 13, but additional studies are needed to assess the efficacy of these bacteriocins within food matrices or *in vivo*.

Results from this study have shown for the first time that *blpU* from strain B59671 encodes a functional bacteriocin, BlpU, with unique broad-spectrum antimicrobial activities against *P. acidilactici* PAF, *L. innocua*, and *S. mutans* UA159. The unsuccessful attempts to knock out *blpU* in B59671 suggested it could have an essential function in this strain, or that the cloning strategy may have impaired the immunity protein encoded by *orf3*. With the immunity protein for BlpK currently unknown, the possibility exists that *orf3* is required for host immunity against both BlpU and BlpK. Mutants resulting in the deletion of *blpU* were obtained in strain LMD-9 (Fontaine and Hols, 2008), but this may have been possible due to the substantial differences between the *blp* gene clusters in LMD-9 and B59671, specifically the absence of a BlpK homolog in LMD-9. Further studies are required to determine if BlpK can function as an independent broad-spectrum bacteriocin, similar to what was shown for BlpU in this study.

## Data availability statement

The genome sequence for S. thermophilus B59671, which includes the blp gene cluster shown, is deposited in GenBank with the accession number CP022547.

## Author contributions

JR: Conceptualization, Data curation, Formal analysis, Investigation, Methodology, Project administration, Supervision, Writing – original draft, Writing – review & editing. GS: Conceptualization, Formal analysis, Methodology, Project administration, Supervision, Writing – original draft, Writing – review & editing. PQ: Methodology, Formal analysis, Writing –review & editing. DS: Data curation, Formal analysis, Writing – original draft, Writing – review & editing. MM: Formal analysis, Methodology, Writing – review & editing. AM: Formal analysis, Methodology, Writing – review & editing. GG: Writing – review & editing. AO: Data curation, Formal analysis, Methodology, Writing – review & editing.

## Supplementary material

SUPPLEMENTARY FIGURE S1 RP-HPLC chromatograph for synthesized BlpU peptide (Peak 4) from *S. thermphilus* B59671 (LifeTein, Hillsborough, NJ). Column: 4.6 × 250 mm, PLRP-S 100A; Mobile Phase: A=0.1% TFA/Acetonitrile, B = 0.1% TFA/water; Flow rate: 1.0 mL/min, UV wavelength: 220 nm; injection volume was 5 μL.
