# Supplementary figures and images for: BlpU is a broad-spectrum bacteriocin in Streptococcus thermophilus
Source: Front Microbiol. 2024 Jul 16;15:1409359. doi: 10.3389/fmicb.2024.1409359 (PMC11286413; doi:10.3389/fmicb.2024.1409359)

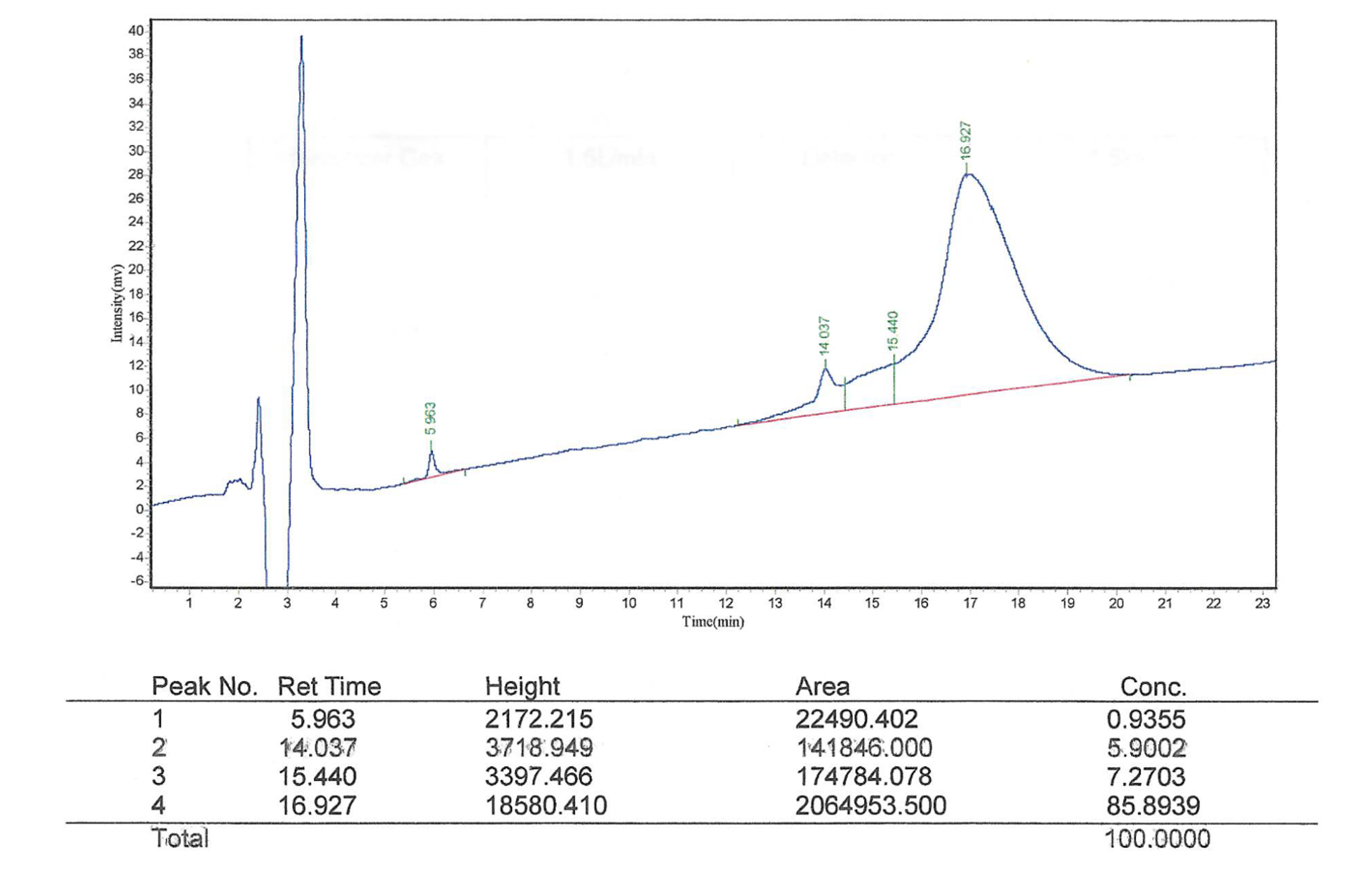

Supplement: SUPPLEMENTARY FIGURE S1 — RP-HPLC chromatograph for synthesized BlpU peptide (Peak 4) from S. thermphilus B59671 (LifeTein, Hillsborough, NJ). Column: 4.6 × 250 mm, PLRP-S 100A; Mobile Phase: A=0.1% TFA/Acetonitrile, B = 0.1% TFA/water; Flow rate: 1.0 mL/min, UV wavelength: 220 nm; injection volume was 5 μL. [file Image_1.TIF]
